# ^1^H NMR Metabolomics of Chinese Human Milk at Different Stages of Lactation among Secretors and Non-Secretors

**DOI:** 10.3390/molecules27175526

**Published:** 2022-08-27

**Authors:** Maaria Kortesniemi, Tahereh Jafari, Yumei Zhang, Baoru Yang

**Affiliations:** 1 Food Sciences, Department of Life Technologies, University of Turku, FI-20014 Turku, Finland; 2 Department of Nutrition and Food Hygiene, Peking University, Xueyuan Road 38, Haidian District, Beijing 100191, China

**Keywords:** ^1^H NMR, human milk, human milk oligosaccharides, lactation, metabolomics, secretor

## Abstract

Human milk is an intricate, bioactive food promoting infant health. We studied the composition of human milk samples collected over an 8-month lactation using ^1^H NMR metabolomics. A total of 72 human breast milk samples were collected from ten Chinese mothers at eight different time points. The concentrations of ten human milk oligosaccharides (HMOs), fucose and lactose were quantified. Six of the mothers were classified as Lewis-positive secretors (Se^+^Le^+^) and four as Lewis-positive non-secretors (Se^−^Le^+^) based on the levels of 2′-fucosyllactose (2′-FL), lacto-N-fucopentaose (LNFP) II, lactodifucotetraose (LDFT) and lacto-N-neotetraose (LNnT). Acetate, citrate, short/medium-chain fatty acids, glutamine and urea showed a time-dependent trend in relation to the stage of lactation. The concentrations of 2′-FL, 3-FL (3-fucosyllactose), 3′-SL (3′-sialyllactose), LDFT, LNFP I, LNFP II, LNFP III, LNnT, LNT (lacto-N-tetraose), and fucose were statistically different between secretors and non-secretors. A temporal difference of approximately 1–2 months between the development of non-secretor and secretor HMO profiles was shown. The results highlighted the importance of long-term breastfeeding, especially among non-secretors.

## 1. Introduction

Human milk is the optimal nutrition for infants. Exclusive breastfeeding is generally recommended during the first six months of life [1]. Human milk contains important macro- and micronutrients, immunological components, and bioactive proteins and oligosaccharides for promoting infant growth and development [2,3]. The composition of human milk exhibits biochemical variability in relation to the mother’s phenotype, diet, disease, and lifestyle [4,5]. The levels of many of the small molecules, including human milk oligosaccharides (HMOs), are also associated with the stage of lactation [5].

Lactation can be divided into different stages based on the milk composition, with the cutoff points being, for example, ≤5 days postpartum, 6–15 days postpartum, and ≥15 days postpartum for colostrum, transitional milk and mature milk, respectively [6]. Metabolomics has been used to follow the compositional changes of human milk over the course of lactation [7,8,9,10]. However, studies on human milk metabolites beyond the recommended breastfeeding duration of 6 months are relatively scarce [11,12,13,14].

The HMOs, based on glucose, galactose, and *N*-acetylglucosamine with varying terminal fucose and sialic acid linkages, are the third largest compound group after lactose and lipids [15]. The HMO profile is determined by genetic factors, gestational age and stage of lactation [7,16]. Fucosyltransferases are enzymes catalyzing the synthesis of HMOs in the mammary gland [17]. Mothers can be classified as secretors or non-secretors based on the expression level of fucosyltransferase 2 (FUT2) encoded by the *Secretor* (*Se*) gene [18]. FUT2 produces *α*1,2-fucosylated HMOs, such as 2′-fucosyllactose (2′-FL), the most abundant HMO in secretor milk [18]. Fucosyltransferase 3 (FUT3) encoded by the *Lewis* (*Le*) gene determines the *α*1,3/4-fucosyltransferase activity [17]. The Chinese population has a unique distribution of *Secretor* and *Lewis* polymorphisms, which affect the HMO profile and total HMO concentration [14,19].

HMOs are known to exhibit immunomodulatory effects and prebiotic properties [20,21]. HMOs are not digested in the infant but are utilized by specific bacteria [22]. HMOs have many beneficial functions in the digestive tract of infants, such as acting against pathogens, increasing the abundance of bifidobacteria, modulating epithelial and mucosal barrier and affecting immune cell function [21,22].

The aim of this research was to study the metabolic profiles of human milk samples collected at eight different time points of lactation up to 8 months postpartum. The secretor status of ten Chinese mothers was studied using ^1^H NMR metabolomics, with a targeted analysis of selected human milk oligosaccharides (HMOs) and sugars.

## 2. Results

**Figure 1:**
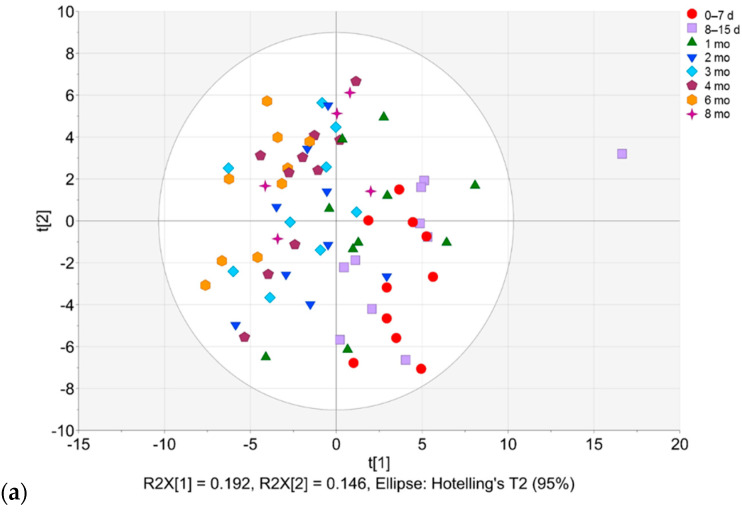

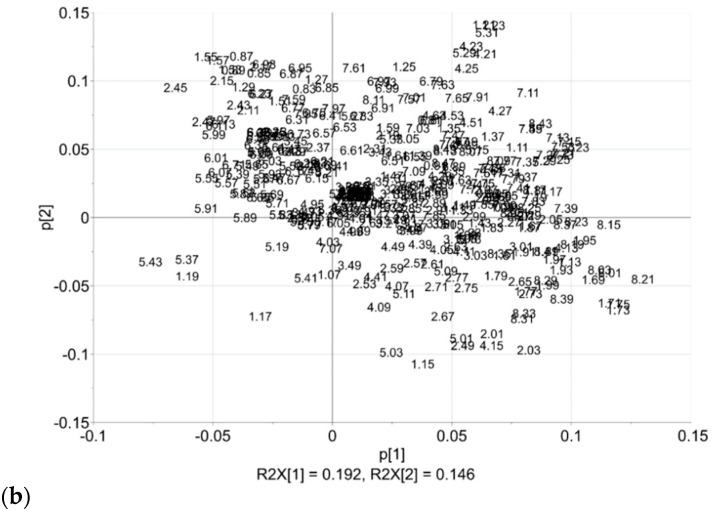
Principal component analysis (PCA) based on binned NMR data (72 observations, 375 *X*-variables, *R*^2^*X*_(cum)_ = 0.741, *Q*^2^_(cum)_ = 0.499). (**a**) Scores plot. Scores colored according to the time point (red circles, 0–7 d (days); lavender boxes, 8–15 d; green upward triangles, 1 mo (month); blue downward triangles, 2 mo; sky blue diamonds, 3 mo; plum pentagons, 4 mo; orange hexagons, 6 mo; purple stars = 8 mo). (**b**) Loadings plot.

Principal component analysis (PCA) was performed on the binned NMR spectra (Figure 1). The first two components explained 33.8% of the total variance. The lactational age-related trend was observed along the first component. The milk samples collected between the time points between 0–7 days and 1 month were mostly located on the positive half of PC1, while the samples from 2 to 8 months were located on the negative half of PC1.

2′-Fucosyllactose (2′-FL; bins 1.21, 1.23, 4.21–4.25 and 5.31) gave strong loadings on the upper right quadrant, and bin 8.15, 3′-galactosyllactose (3′-GL; bins 2.03, 4.15 and 8.21), 6′-sialyllactose (6′-SL; bins 1.73, 1.75, 8.01 and 8.03) and lacto-N-fucopentaose II (LNFP II; bins 1.15, 5.01 and 5.03), citrate (bin 2.49, 2.67 and 2.71) and acetate (bin 1.91, 1.93) on the lower right quadrant. Short-/medium-chain fatty acids (bins 0.85–0.89, 1.53–1.57, and 2.15) and glutamine (bin 2.45) gave strong loadings on the upper left quadrant. 3-FL (bins 1.19, 5.37, 5.43), urea (bins 5.89 and 5.91) and bin 1.17 (contributing compounds: 3-FL, LNFP II, lacto-N-fucopentaose III (LNFP III) and ethanol) gave strong loadings near the border of the upper and lower left quadrants (PC1(−)). The PCA model (Figure 1a) revealed an outlier (time point 8–15 days), which was explained by a relatively high level of phenylalanine.

**Figure 2:**
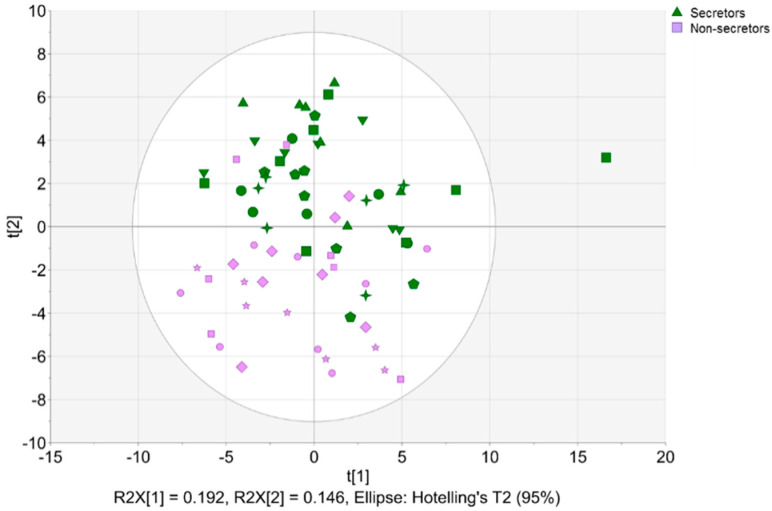
Principal component analysis (PCA) based on binned NMR data (72 observations, 375 *X*-variables; *R*^2^*X*_(cum)_ = 0.741, *Q*^2^_(cum)_ = 0.499). Scores plot colored according to secretor status (green symbols, secretors; lavender symbols, non-secretors), with individual mothers marked with different symbols.

Based on the HMO profiles, six out of the ten mothers were categorized as secretors (Se^+^) and four as non-secretors (Se^−^) (Figure 2). The human milk samples of the non-secretor mothers were characterized by low concentration of 2′-FL (<0.08 mmol/L) and lacto-N-fucopentaose I (LNFP I; <0.05 mmol/L), relatively high concentration of 3-fucosyllactose (3-FL), and the absence of lacto-N-tetraose (LNT) and lactodifucotetraose (LDFT). Further, all mothers were specified as Lewis-positive (Se^+^Le^+^/Se^−^Le^+^) based on the presence of LNFP II [23]. 

**Figure 3:**
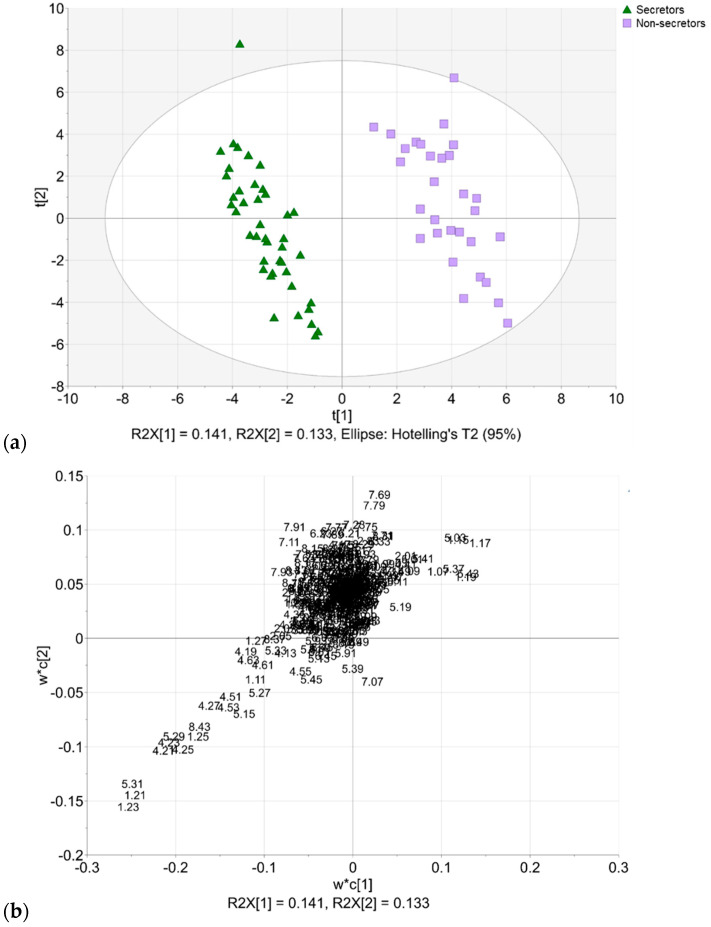
Partial least squares discriminant analysis (PLS-DA) based on binned NMR data (72 observations, 375 *X*-variables, *R*^2^*X*_(cum)_ = 0.387, *R*^2^*Y*_(cum)_ = 0.976, *Q*^2^_(cum)_ = 0.945). (**a**) Scores plot. Scores colored according to secretor status (green upward triangles, secretors; lavender boxes, non-secretors). (**b**) Loadings plot.

The secretor and non-secretor samples partly overlapped in the PCA model (Figure 2). Once the partial least squares discriminant analysis (PLS-DA) was applied, a full discrimination of the sample groups was observed (Figure 3).

**Figure 4:**
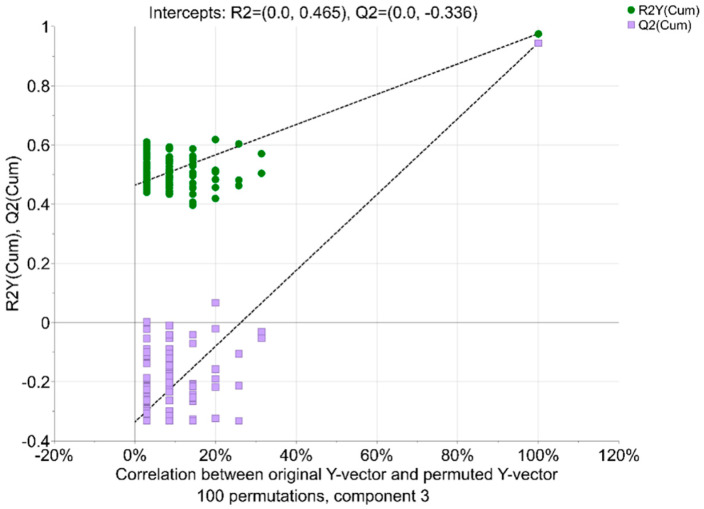
Permutation plot for PLS-DA validation.

Bins attributed to 2′-FL (1.21, 1.23, 1.25, 4.21, 4.53, 4.25, 4.27, 5.29, and 5.31) explained the coordinates for the secretor samples. The bins attributed to LNFP III (1.15, 1.17, and 2.03), LNFP II (1.17, 2.03, and 5.03), and 3′-FL (1.19, 5.19, 5.37, and 5.43) explained the coordinates for the non-secretor samples. The validation of the three-component PLS-DA model was performed using a permutation test and CV-ANOVA (Figure 4). The *Y*-intercept for *R*^2^*Y* was slightly higher than recommended for a model validity (<0.3–0.4), however, the *Y*-intercept for *Q*^2^ was valid (<0.05) [24]. The CV-ANOVA returned *F* of 153.104 and *p*_CV-ANOVA_ < 0.05, denoting a significant model.

**Figure 5:**
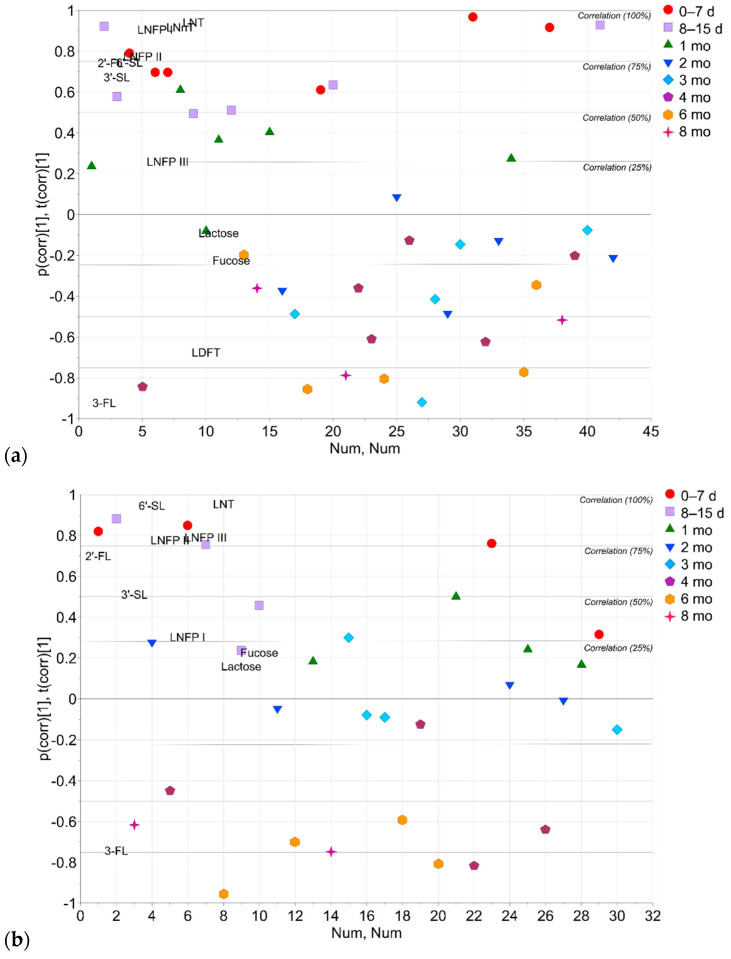
Principal component analysis (PCA) based on the concentrations of HMOs, lactose and fucose. (**a**) Biplot, secretors (PC1 vs. index number; 42 observations, 12 *X*-variables; *R*^2^*X*[1] = 0.647, *Q*^2^[1] = 0.563). (**b**) Biplot, non-secretors (PC1 vs. index number; 30 observations, 10 *X*-variables; *R*^2^*X*[1] = 0.569, *Q*^2^[1] = 0.448). Scores colored according to the lactation time point (red circles, 0–7 d (days); lavender boxes, 8–15 d; green upward triangles, 1 mo (month); blue downward triangles, 2 mo; sky blue diamonds, 3 mo; plum pentagons, 4 mo; orange hexagons, 6 mo; purple stars, 8 mo). Abbreviations: 2′-FL, 2′-fucosyllactose; 3-FL, 3-fucosyllactose; 3′-SL, 3-sialyllactose; 6′-SL, 6′-sialyllactose; LDFT, lactodifucotetraose; LNFP I, lacto-N-fucopentaose I; LNFP II, lacto-N-fucopentaose II; LNFP III, lacto-N-fucopentaose III; LNnT, lacto-N-neotetraose; LNT, lacto-N-tetraose.

PCA models based on the concentrations of 12 targeted compounds 2′-FL, 3-FL, 3′-sialyllactose (3′-SL), 6′-SL, LDFT, LNFP I, LNFP II, LNFP III, lacto-N-neotetraose (LNnT), LNT, fucose and lactose were built separately for secretors and non-secretors (Figure 5). The models included only one principal component, but the PCAs showed a temporal trend in the HMO concentrations. In the secretor model (Figure 5a), the samples from the first three time points were mainly located above the +25% correlation line on the positive half of PC1. In the non-secretor model (Figure 5b), the time points 4–8 months were located below the −25% correlation line on the negative half of the PC1. The PCAs in Figure 5 show the temporal dynamics of the HMO profiles, e.g., the decrease of 2′-FL and the increase of 3-FL over the course of lactation (inverse correlation). In Figure 5a, LNFP I shows a positive correlation with LNFP II, LNT and LNnT, whereas in Figure 5b LNFP I shows a correlation with lactose and fucose. Both models in Figure 5 have a region where the scores are not overlapping significantly. In Figure 5a, most of the scores representing the time points from 0–7 days to 1 month are located above the +20% correlation on the positive half of the PC1, while the most of the time points representing the time points from 2 months to 8 months are below the −10% correlation on the negative half of the PC1. In Figure 5b, most of the scores representing the time points up from 0–7 days to 3 months are located above the −20% correlation, and most of the score representing time points from 4 months to 8 months are located below the −45% correlation.

The concentrations of 2′-FL, 3-FL, 3′-SL, 6′-SL, LDFT, LNFP I, LNFP II, LNFP III, LNnT, LNT, fucose and lactose among the secretors and non-secretors are presented in Table 1. Out of the targeted compounds, only the concentrations of 6′-SL and lactose did not differ statistically significantly between secretors and non-secretors (*p* > 0.05, Mann–Whitney *U* test), whereas the levels of 2′-FL, 3-FL, 3′-SL, LDFT, LNFP I, LNFP II, LNFP III, LNnT, LNT and fucose were statistically different between the two groups (*p* < 0.05, *q* < 0.01). The concentration of 2′-FL ranged from 1.31 mmol/L to 5.78 mmol/L among the secretors and from 3.11 µmol/L to 75.7 µmol/L among non-secretors, the difference between the two groups being highly statistically significant. The content of 3-FL was much higher in the non-secretor milk samples (ranging from 1.29 mmol/L to 4.50 mmol/L) compared to the secretor samples (ranging from 0.22 mmol/L to 1.61 mmol/L).

Based on the mixed-effects model (one-way) for repeated measures, the different stages of lactation were statistically significantly different (*p* < 0.05) in the content of 3-FL, 3′-SL, 6′-SL, LDFT, LNFP II, LNT, and LNnT among secretors, and 3-FL, 6′-SL, LNFP II, and LNT among non-secretors (Table 1). Multiple comparisons of the lactation time points based on the FDR approach [25] revealed the statistical differences regarded as discoveries (*q* < 0.05).

## 3. Discussion

The untargeted metabolomics approach portrayed the holistic changes occurring in human milk during different stages of lactation. The observations based on the PCA model in Figure 1 were consistent with prior reports [7,8]. Only few non-HMO metabolites gave strong loadings on the PCA model (Figure 1). These included acetate, citrate, short/medium-chain fatty acids, glutamine and urea.

The concentration of acetate exhibits a large biological variation in human milk [4]. In a study by Wu et al., no change was observed in the acetate concentration between early (9–24 days) and late (31–87 days) lactation [8]. Here, the acetate concentration was relatively higher in the early stages of lactation. Acetate has been reported to be negatively correlated to human milk Actinobacteria [26]. Citrate levels typically correlate with lactose and 2-oxoglurate [27] and reflect mammary gland activity [28]. Citrate concentration was shown to decrease over the course of lactation as that of acetate.

The levels of short- and medium-chain fatty acids are known to increase with lactational age [7], and similar findings were observed in the current study. The human milk short-chain fatty acids may have an important role in the infant’s growth and development [29,30]. In our previous study [31], a positive association between maternal psychological distress and short- and medium-chain fatty acids in human milk at 2.5 months postpartum was shown, possibly indicating stress-related changes in the mothers’ gut microbiota.

Glutamine, one of the most abundant free amino acids in human milk, was shown to increase with the progression of the lactation stage, as expected [27,32,33]. In addition to the stage of lactation, the gestational age has also been reported to be a factor determining the free glutamine concentration in human milk, being lower in preterm human milk [32]. Lower concentrations of glutamine and glutamate, along with alanine, taurine and betaine in the breast milk of the Lewis-negative non-secretors compared to secretors (but similar between Lewis-positive non-secretors and secretors) have been reported [23].

Our data showed the bins at *δ* 5.89 and 5.91 ppm corresponding to urea correlated with the later stages of lactation (PC1(−), Figure 1). Although urea may be affected by the water suppression due to the chemical exchange of protons, a similar trend in human milk urea concentrations determined with NMR has been reported earlier [4,34]. A positive correlation of urea with Actinobacteria in the human milk microbiota has been shown [26].

Lactose is the main carbohydrate in milk, constituting around 2–8% of milk [15]. Lactose concentration is relatively constant after the colostrum phase [11]. Despite the high biological variation [4], fucose concentrations seemed to increase over time. However, there was no statistically significant difference between the time points representing different lactation stages (*p* > 0.05, mixed-effects model (one-way) for repeated measures).

The HMO data reported in literature vary greatly depending on the methods, standard compounds and libraries used [4,16,35,36,37]. In general, the total concentration of HMOs decreases over lactation [8,35]. As 3′-GL decreases with lactational age, in this study, 3′-GL was not detected in the human milk samples collected after 1 month postpartum. It is possible that 3′-GL and other galactosyllactoses may have anti-inflammatory properties [38].

The prevalence of secretors in the Chinese population is approximately 50–70% [18]. The total HMO concentration is typically lower among non-secretors compared to secretors, regardless of the stage of lactation [36]. All mothers in the present study were deemed Lewis-positive (Le^+^), producing HMOs such as 3-FL, LNFP II and LNFP III [35]. The concentrations of 2′-FL, 3-FL, 3′-SL, LDFT, LNFP I, LNFP II, LNFP III, LNT, LNnT and fucose were statistically significant between secretors and non-secretors (*p* < 0.05, Mann–Whitney *U*). In our previous study with Finnish mothers, secretor and non-secretor milk did not differ in 3′-SL and LNFP III [31]. In a multi-country study, Chinese human milk samples (at 1 month postpartum) contained significantly higher levels of 3-FL and LNFP III compared to Finnish samples [26].

The concentration of 2′-FL is relatively lower in the milk of Chinese secretors compared to Western populations [39]. In a study by Wang et al., the concentration limit of 2′-FL for secretor status was established at 15 mg/L among Chinese participant mothers [16]. Low concentrations of 2′-FL were also present in the Chinese non-secretor milk [35]. Here, the 2′-FL concentrations in the non-secretor milk ranged from 0.01 mmol/L to 0.05 mmol/L. In addition, the α1,2-linked LNFP I was present in the non-secretor milk (0.02 ± 0.01 mmol/L). The presence of 2′-FL and LNFP I in the non-secretor milk is consistent with prior research suggesting that Chinese non-secretors have some FUT2 enzyme activity [35]. This is likely owing to a *se* allele encoding a weak FUT2 with reduced activity, as in Japanese populations [40].

Although the concentrations of 2′-FL appeared to decrease over time, the statistical difference between the time points was not significant. However, the concentration of 3-FL increased over the course of lactation. 2′-FL and 3-FL are suggested to be co-regulated on corresponding fucosyltransferases or compete on the shared substrate, GDP-l-fucose [17]. 3-FL is synthesized through the action of the *Le* gene, while FUT3 is the key enzyme for 3-FL biosynthesis [17]. 3-FL can selectively promote the growth of bifidobacteria. A delay in the colonization of gut bifidobacteria in the infants of non-secretor mothers has been reported [41]. Based on how the human milk samples from different time points clustered in the PCAs in Figure 5, there seemed to be a stretch between the time points 1 month and 2 months (Figure 5a) in the secretor group, and between 3 months and 4 months (Figure 5b) in the non-secretor group. This observation would deserve more research with a larger statistical sample, as well as to further investigate whether this difference is associated with the delay in the colonization of gut bifidobacteria.

Bifidobacteria and Bacteroides have been identified as major HMO consumers in the infant gut [41]. The formation of bifidobacteria-rich microbiota in the infant gut is associated with the HMO uptake by fucosyllactose transporter-2, characterized e.g., from *Bifidobacterium longum* ssp. *infantis* [42]. According to the study by Korpela et al. [43], the sequence-level diversity of both bifidobacteria and Bacteroides is lower in the gut microbiota of infants born by cesarean to non-secretor mothers. The gut microbiota of such infants is also characterized by increased enterococci [43]. In addition to the prebiotic properties, 3-FL has been confirmed to have immunomodulatory, antiadhesive antimicrobial, antiviral, and gastrointestinal protective properties, as reviewed by Li et al. [17]. Here, the concentration of 3-FL ranged from 0.12 mmol/L (stage of lactation 0–7 days) to 1.81 mmol/L (8 months) among the secretors and from 0.96 mmol/L (0–7 days) to 5.86 mmol/L (4 months) among the non-secretors. The higher concentration of 3-FL among non-secretors compared to secretors is compliant with prior reports [4,35]. The concentrations of 3-FL during the first three lactation time points among the non-secretors (Table 1) were similar to those reported in human milk collected at 0–5 days, 14 days and 28 days from secretor and non-secretor mothers in the US [34].

LNnT and LDFT were not detected in the non-secretor milk. LNT has been reported to be present in Chinese human milk (including non-secretors) at concentrations above 21 mg/kg [35]. The concentration of LNT was higher among the non-secretors (*p* < 0.0001, *q* < 0.0001; Mann–Whitney *U*). Preterm delivery is generally associated with variations in LNT concentration [44]. In particular, the strains of *Bifidobacterium breve* can utilize non-fucosylated oligosaccharides LNT and LNnT [45]. LDFT, present only in the secretor samples, increased over the course of lactation up to the 4-month time point, after which it remained stable. In a study by Xu et al., the concentration of LDFT, however, remained unchanged during the lactation of 10–120 days postpartum observed for US mothers [36]. Although Austin et al. studied the HMO composition up to 8 months postpartum as in the current study, LDFT was not included in their analysis [35]. Here, the LDFT concentrations were statistically significantly different between the time points 8–15 days and 3 months, 8–15 days and 4 months, as well as 8–15 days and 6 months (*q* < 0.05, mixed-effects model (one-way) for repeated measures). LDFT is positively correlated with other α-1,2-fucosylated HMOs, 2′-FL, LNFP-I, lacto-N-difucohexaose-I (LNDFH-I), difucosyllacto-N-hexaose a (DFLNHa) [14], and it has been reported to significantly attenuate TNF-α induced inflammation in immature intestinal epithelial cells in vitro [46]. A similar effect has also been detected with 3-FL and LNnT [46].

## 4. Materials and Methods

### 4.1. Human Milk Sample Collection

A total of 72 human milk samples were included in this study. The milk samples were collected from Chinese mothers (*n* = 10) of the Beijing area at eight different lactational time points: 0–7 days (*n* = 10), 8–15 days (*n* = 10), 1 month (*n* = 10), 2 months (*n* = 9), 3 months (*n* = 9), 4 months (*n* = 10), 6 months (*n* = 9) and 8 months (*n* = 5). The sample collection was conducted according to the guidelines in the Declaration of Helsinki, with the approval of the Medical Ethics Research Board of Peking University (Ref: IRB00001052-16038). Written informed consents were obtained from all participant mothers.

The human milk samples were kept frozen at −20 °C until they were delivered to the laboratory and subsequently stored at −80 °C. The samples were stored in dry ice upon transportation to Finland and stored at −80 °C until analysis.

### 4.2. NMR Spectroscopy

The human milk samples were prepared and analyzed according to the method by Smilowitz et al. [4] with minor modifications [31]. The filtered milk sample was combined with 10% of a Chenomx internal standard solution containing 3-(trimethylsilyl)-1-propanesulfonic acid-*d*_6_ sodium salt (DSS-*d*_6_) and 0.03% sodium azide (NaN_3_) in D_2_O, and 180 µL of the sample was placed in a 3-mm NMR tube for analysis. ^1^H NMR spectra were acquired on a Bruker Avance 600 MHz NMR spectrometer equipped with a nitrogen-cooled PRODIGY TCI-cryoprobe and a SampleJet autosampler. The spectra were collected using a NOESY-presaturation pulse sequence (*noesygppr1d*) with 512 transients, 4 dummy scans, a spectral width of 14 ppm, an acquisition time of 3.9 s, a relaxation delay (d1) of 5.0 s, and a mixing time (d8) of 100 ms. The Fourier-transformed spectra were phase-, baseline-, and shim-corrected (to 0.9 Hz) in a Chenomx Processor (Chenomx NMR Suite 8.3, Chenomx Inc., Edmonton, AB, Canada).

### 4.3. Data Analysis

The NMR signals were assigned based on an in-house standard compound library (Chenomx Profiler) and literature. The metabolites were quantified in reference to the internal standard (DSS-*d*_6_) in the Chenomx Profiler. The maternal secretor status affecting the human milk oligosaccharide (HMO) composition was assessed based on the 2′-fucosyllactose (2′-FL) resonances in the spectra [7].

The NMR data was normalized relative to the total area under each spectrum line and binned with 0.02 ppm bin size (Chenomx). Bins 0.81–8.49 were included in the data analysis with the exclusion of the water region. The binned data was log-transformed and Pareto-scaled in SIMCA 16 (Sartorius Stedim Data Analytics AB, Sweden) prior to multivariate data analyses, principal component analysis (PCA) and partial-least squares discriminant analysis (PLS-DA). The validation of the PLS-DA model was performed using a permutation test (100 permutations) and CV-ANOVA.

Twelve sugars and small soluble milk glycans, i.e., lactose, fucose, 2′-fucosyllactose (2′-FL), 3-fucosyllactose (3-FL), 3′-sialyllactose (3′-SL), 6′-sialyllactose (6′-SL), lactodifucotetraose (LDFT), lacto-N-fucopentaose I, lacto-N-fucopentaose II, lacto-N-fucopentaose III, lacto-N-tetraose (LNT), and lacto-N-neotetraose (LNnT) were quantified with the Chenomx Profiler. The data was log-transformed and Pareto-scaled in SIMCA 16 prior to multivariate data analysis (PCA).

Statistical analyses were performed with GraphPad Prism v9.4.0 (GraphPad Software, Inc., San Diego, CA, USA). An assessment of normality was performed using the Shapiro–Wilk test. The non-parametric Mann–Whitney *U* test was used to test the statistical differences in the targeted metabolites between secretors and non-secretors. The two-stage linear step-up procedure of Benjamini, Krieger and Yekutieli (Q: 5%) was used as the false discovery rate method.

To compare the concentrations at different stages of lactation, the data values were log-transformed to correct non-normal distribution and analyzed by fitting a mixed-effects model (one-way) for repeated measures with the Geisser–Greenhouse correction (sphericity not assumed), suitable for data containing missing values. The *p*-values were corrected for multiple comparisons by controlling the false discovery rate (the two-stage linear step-up procedure of Benjamini, Krieger and Yekutieli, Q: 5%) [25].

## 5. Conclusions

We used untargeted and targeted ^1^H NMR metabolomics to analyze the composition of 72 human milk samples collected from 10 Chinese mothers at eight stages of lactation from the colostrum stage (0–7 days) until 8 months postpartum. Our results showed a temporal delay of approximately 1–2 months in the development non-secretor HMO profile compared to secretor milk. The pivotal changes in the HMO profile occurred between 1 and 2 months in secretors, and between 3 and 4 months in non-secretors. The results highlight the importance of exclusive breastfeeding during the recommended 6 months postpartum, especially among non-secretors, for the infant to gain the benefits of the HMOs.

## Figures and Tables

**Table 1:** Concentrations (mean ± standard deviation; mmol/L) of selected human milk oligosaccharides, fucose and lactose across different stages of lactation and among secretors and non-secretors.

	Time Point	Secretors	Non-Secretors	*p* (*q*) ^1^
**2′-fucosyllactose**	0–7 d	4.28 ± 0.68 (*n* = 6)	0.04 ± 0.01 (*n* = 4)	
	8–15 d	4.39 ± 0.47 (*n* = 6)	0.05 ± 0.01 (*n* = 4)	
	1 mo	4.15 ± 0.82 (*n* = 6)	0.04 ± 0.02 (*n* = 4)	
	2 mo	3.41 ± 0.91 (*n* = 5)	0.05 ± 0.01 (*n* = 4)	
	3 mo	2.98 ± 0.53 (*n* = 5)	0.03 ± 0.01 (*n* = 4)	
	4 mo	2.79 ± 0.76 (*n* = 6)	0.01 ± 0.01 (*n* = 4)	
	6 mo	2.50 ± 0.48 (*n* = 5)	0.02 ± 0.01 (*n* = 4)	
	8 mo	2.02 ± 0.62 (*n* = 3)	0.02 ± 0.02 (*n* = 2)	
		0.2884 ^2^	0.0838	
	all	3.44 ± 1.02 (*n* = 42)	0.03 ± 0.02 (*n* = 30)	<0.0001 (<0.0001)
**3-fucosyllactose**	0–7 d	0.22 ± 0.08	1.29 ± 0.34	
	8–15 d	0.36 ± 0.14	1.77 ± 0.35	
	1 mo	0.54 ± 0.23	2.19 ± 0.32	
	2 mo	0.81 ± 0.34	3.35 ± 0.78	
	3 mo	0.99 ± 0.22	3.24 ± 0.94	
	4 mo	1.29 ± 0.41	4.00 ± 1.27	
	6 mo	1.20 ± 0.23	3.22 ± 0.99	
	8 mo	1.61 ± 0.19	4.50 ± 0.24	
		<0.0001 ^3^	0.0023 ^4^	
	all	0.81 ± 0.50	2.84 ± 1.21	<0.0001 (<0.0001)
**3′-sialyllactose**	0–7 d	0.16 ± 0.04	0.20 ± 0.05	
	8–15 d	0.18 ± 0.03	0.17 ± 0.04	
	1 mo	0.11 ± 0.02	0.13 ± 0.02	
	2 mo	0.09 ± 0.02	0.13 ± 0.04	
	3 mo	0.09 ± 0.01	0.13 ± 0.05	
	4 mo	0.10 ± 0.02	0.13 ± 0.03	
	6 mo	0.10 ± 0.02	0.10 ± 0.05	
	8 mo	0.11 ± 0.01	0.12 ± 0.04	
		0.0010 ^5^	0.1133	
	all	0.12 ± 0.04	0.14 ± 0.05	0.0337 (0.0071)
**6′-sialyllactose**	0–7 d	0.49 ± 0.23	0.70 ± 0.24	
	8–15 d	0.49 ± 0.24	0.60 ± 0.35	
	1 mo	0.36 ± 0.18	0.44 ± 0.07	
	2 mo	0.23 ± 0.17	0.26 ± 0.11	
	3 mo	0.13 ± 0.06	0.24 ± 0.16	
	4 mo	0.13 ± 0.02	0.10 ± 0.05	
	6 mo	0.07 ± 0.03	0.05 ± 0.01	
	8 mo	0.08 ± 0.03	0.07 ± 0.03	
		0.0027 ^6^	0.0068 ^7^	
	all	0.27 ± 0.22	0.32 ± 0.28	0.6621 (0.1159)
**LDFT**	0–7 d	0.15 ± 0.06	n.d.	
	8–15 d	0.14 ± 0.03	n.d.	
	1 mo	0.18 ± 0.09	n.d.	
	2 mo	0.21 ± 0.06	n.d.	
	3 mo	0.24 ± 0.05	n.d.	
	4 mo	0.27 ± 0.06	n.d.	
	6 mo	0.27 ± 0.06	n.d.	
	8 mo	0.27 ± 0.07	n.d.	
		0.0302 ^8^	n.a.	
	all	0.21 ± 0.08	0.00 ± 0.00	<0.0001 (<0.0001)
**LNFP I**	0–7 d	1.07 ± 0.43	0.03 ± 0.01	
	8–15 d	0.94 ± 0.41	0.02 ± 0.01	
	1 mo	0.49 ± 0.28	0.02 ± 0.01	
	2 mo	0.19 ± 0.12	0.02 ± 0.00	
	3 mo	0.16 ± 0.11	0.03 ± 0.01	
	4 mo	0.13 ± 0.10	0.02 ± 0.01	
	6 mo	0.11 ± 0.09	0.01 ± 0.00	
	8 mo	0.11 ± 0.08	0.02 ± 0.01	
		0.1295	0.1503	
	all	0.44 ± 0.45	0.02 ± 0.01	<0.0001 (<0.0001)
**LNFP II**	0–7 d	0.15 ± 0.05	0.66 ± 0.42	
	8–15 d	0.18 ± 0.07	0.82 ± 0.29	
	1 mo	0.14 ± 0.08	0.48 ± 0.20	
	2 mo	0.07 ± 0.01	0.41 ± 0.20	
	3 mo	0.06 ± 0.04	0.40 ± 0.09	
	4 mo	0.08 ± 0.02	0.25 ± 0.14	
	6 mo	0.05 ± 0.04	0.21 ± 0.07	
	8 mo	0.07 ± 0.01	0.23 ± 0.06	
		0.0043 ^9^	0.0726	
	all	0.10 ± 0.06	0.44 ± 0.29	<0.0001 (<0.0001)
**LNFP III**	0–7 d	0.17 ± 0.07	0.32 ± 0.13	
	8–15 d	0.15 ± 0.07	0.34 ± 0.18	
	1 mo	0.15 ± 0.08	0.24 ± 0.09	
	2 mo	0.12 ± 0.02	0.27 ± 0.06	
	3 mo	0.12 ± 0.04	0.23 ± 0.04	
	4 mo	0.14 ± 0.04	0.16 ± 0.05	
	6 mo	0.13 ± 0.06	0.13 ± 0.05	
	8 mo	0.13 ± 0.04	0.17 ± 0.02	
		0.4247	0.0203 ^10^	
	all	0.14 ± 0.06	0.24 ± 0.11	<0.0001 (<0.0001)
**LNT**	0–7 d	0.82 ± 0.25	1.73 ± 0.84	
	8–15 d	0.73 ± 0.41	1.26 ± 0.44	
	1 mo	0.40 ± 0.27	0.65 ± 0.21	
	2 mo	0.15 ± 0.07	0.37 ± 0.14	
	3 mo	0.17 ± 0.10	0.39 ± 0.16	
	4 mo	0.12 ± 0.08	0.21 ± 0.12	
	6 mo	0.10 ± 0.05	0.14 ± 0.11	
	8 mo	0.14 ± 0.09	0.12 ± 0.06	
		0.0021 ^11^	0.0103 ^12^	
	all	0.36 ± 0.35	0.64 ± 0.65	<0.0001 (<0.0001)
**LNnT**	0–7 d	0.23 ± 0.04	n.d.	
	8–15 d	0.18 ± 0.04	n.d.	
	1 mo	0.11 ± 0.05	n.d.	
	2 mo	0.05 ± 0.02	n.d.	
	3 mo	0.04 ± 0.02	n.d.	
	4 mo	0.04 ± 0.03	n.d.	
	6 mo	0.05 ± 0.03	n.d.	
	8 mo	0.05 ± 0.03	n.d.	
		0.0030 ^13^	n.a.	
	all	0.10 ± 0.08	0.00 ± 0.00	<0.0001 (<0.0001)
**Fucose**	0–7 d	0.26 ± 0.17	0.06 ± 0.03	
	8–15 d	0.22 ± 0.12	0.03 ± 0.01	
	1 mo	0.30 ± 0.14	0.03 ± 0.01	
	2 mo	0.30 ± 0.16	0.03 ± 0.02	
	3 mo	0.37 ± 0.18	0.04 ± 0.03	
	4 mo	0.39 ± 0.13	0.04 ± 0.02	
	6 mo	0.41 ± 0.19	0.03 ± 0.02	
	8 mo	0.45 ± 0.02	0.04 ± 0.02	
		0.0599	0.5847	
	all	0.33 ± 0.16	0.04 ± 0.02	<0.0001 (<0.0001)
**Lactose**	0–7 d	129.67 ± 7.63	131.88 ± 23.42	
	8–15 d	147.74 ± 7.82	149.28 ± 2.35	
	1 mo	156.56 ± 6.22	146.22 ± 9.12	
	2 mo	146.66 ± 20.55	153.33 ± 11.25	
	3 mo	144.73 ± 19.29	148.36 ± 7.01	
	4 mo	152.79 ± 8.04	152.86 ± 6.72	
	6 mo	143.96 ± 14.73	126.42 ± 31.89	
	8 mo	154.76 ± 11.05	143.31 ± 6.55	
		0.1206	0.2986	
	all	146.70 ± 14.15	144.00 ± 16.98	0.5137 (0.0981)

^1^ Exact, two-tailed *p*-value (Mann–Whitney *U*); *q*-value (in parentheses; two-stage step-up procedure of Benjamini, Krieger and Yekutieli, Q: 5%). ^2^ *p*-value (mixed-effects model (one-way) for repeated measures). ^3^ Multiple comparisons *q* < 0.05 (3-FL, secretors): all excluding 4 mo vs. 6 mo, 4 mo vs. 8 mo, 6 mo vs. 8 mo. ^4^ Multiple comparisons *q* < 0.05 (3-FL, non-secretors): 0–7 d vs. 1 mo, 0–7 d vs. 2 mo, 0–7 d vs. 3 mo, 0–7 d vs. 4 mo, 0–7 d vs. 6 mo, 8–15 d vs. 2 mo, 8–15 d vs. 3 mo, 8–15 d vs. 4 mo, 8–15 d vs. 6 mo, 1 mo vs. 2 mo, 1 mo vs. 4 mo, 3 mo vs. 4 mo. ^5^ Multiple comparisons *q* < 0.05 (3′-SL, secretors): 8–15 d vs. 1 mo, 8–15 d vs. 3 mo. ^6^ Multiple comparisons *q* < 0.05 (6′-SL, secretors): 0–7 d vs. 1 mo, 0–7 d vs. 3 mo, 0–7 d vs. 4 mo, 0–7 d vs. 6 mo, 0–7 d vs. 8 mo, 8–15 d vs. 3 mo, 8–15 s vs. 4 mo, 8–15 d vs. 6 mo, 8–15 d vs. 8 mo, 1 mo vs. 3 mo, 1 mo vs. 4 mo, 1 mo vs. 6 mo, 1 mo vs. 8 mo, 2 mo vs. 6 mo, 3 mo vs. 6 mo, 4 mo vs. 6 mo, 4 mo vs. 8 mo. ^7^ Multiple comparisons *q* < 0.05 (6′-SL, non-secretors): 0–7 d vs. 2 mo, 0–7 d vs. 3 mo, 0–7 d vs. 4 mo, 0–7 d vs. 6 mo, 8–15 d vs. 4 mo, 8–15 d vs. 6 mo, 1 mo vs. 4 mo, 1 mo vs. 6 mo, 2 mo vs. 4 mo, 2 mo vs. 6 mo, 3 mo vs. 6 mo. ^8^ Multiple comparisons *q* < 0.05 (LDFT, secretors): 8–15 d vs. 3 mo, 8–15 d vs. 4 mo, 8–15 d vs. 6 mo. ^9^ Multiple comparisons *q* < 0.05 (LNFP II, secretors): none. Multiple comparisons *p* < 0.05: 0–7 d vs. 2 mo, 0–7 d vs. 3 mo, 0–7 d vs. 4 mo, 0–7 d vs. 6 mo, 8–15 d vs. 1 mo, 8–15 d vs. 2 mo, 8–15 d vs. 3 mo, 8–15 d vs. 4 mo, 8–15 d vs. 6 mo, 8–15 d vs. 8 mo, 1 mo vs. 2 mo, 1 mo vs. 3 mo, 1 mo vs. 6 mo, 1 mo vs. 8 mo. ^10^ Multiple comparisons *q* < 0.05 (LNFP III, non-secretors): 0–7 d vs. 6 mo, 2 mo vs. 4 mo. ^11^ Multiple comparisons *q* < 0.05 (LNT, secretors): 0–7 d vs. 1 mo, 0–7 d vs. 2 mo, 0–7 d vs. 3 mo, 0–7 d vs. 4 mo, 0–7 d vs. 6 mo, 0–7 d vs. 8 mo, 8–15 d vs. 1 mo, 8–15 d vs. 2 mo, 8–15 d vs. 3 mo, 8–15 d vs. 4 mo, 8–15 d vs. 6 mo, 8–15 d vs. 8 mo, 1 mo vs. 2 mo, 1 mo vs. 3 mo, 1 mo vs. 4 mo, 1 vs. 6 mo, 1 vs. 8 mo. ^12^ Multiple comparisons *q* < 0.05 (LNT, non-secretors): 0–7 d vs. 1 mo, 0–7 d vs. 2 mo, 0–7 d vs. 3 mo, 0–7 d vs. 4 mo, 0–7 d vs. 6 mo, 8–15 d vs. 1 mo, 8–15 d vs. 2 mo, 8–15 d vs. 3 mo, 8–15 d vs. 4 mo, 8–15 d vs. 6 mo, 8–15 d vs. 8 mo, 1 mo vs. 2 mo, 1 mo vs. 4 mo, 1 mo vs. 6 mo, 1 mo vs. 8 mo, 2 mo vs. 6 mo, 2 mo vs. 8 mo, 3 mo vs. 4 mo, 3 mo vs. 6 mo, 4 mo vs. 6 mo. ^13^ Multiple comparisons *q* < 0.05 (LNnT, secretors): 0–7 d vs. 8–15 d, 0–7 d vs. 1 mo, 0–7 d vs. 2 mo, 0–7 d vs. 3 mo, 0–7 d vs. 4 mo, 0–7 d vs. 6 mo, 8–15 d vs. 2 mo, 8–15 d vs. 3 mo, 8–15 d vs. 4 mo, 8–15 d vs. 6 mo, 8–15 d vs. 8 mo, 1 mo vs. 2 mo, 1 mo vs. 3 mo, 1 mo vs. 4 mo, 1 mo vs. 6 mo, 3 mo vs. 6 mo.

## Data Availability

Not applicable.
